# A Comparison of the Effects of COVID-19 on Irritable Bowel Syndrome and Inflammatory Bowel Disease Patients While Working at Home and in the Office: A Retrospective Study

**DOI:** 10.7759/cureus.33266

**Published:** 2023-01-02

**Authors:** Spyridon Zouridis, Muhammad Farhan Ashraf, Patrick Tempera, Ahmad Abulawi, Umer Ejaz Malik, Hadi Minhas, Asra Batool

**Affiliations:** 1 Medicine, Albany Medical Center, Albany, USA; 2 Gastroenterology, Rochester Regional Health, Rochester, USA; 3 Internal Medicine, Albany Medical Center, Albany, USA; 4 Gastroenterology, Albany Medical Center, Albany, USA

**Keywords:** lifestyle facors and ibd, covid-19, inflammatory bowel disease (ibd), working environment, ibs

## Abstract

Background

Irritable bowel syndrome (IBS) and inflammatory bowel disease (IBD) have a strong relationship with psychological stress. Studies have shown increased stress levels in patients with IBS and IBD during the SARS-CoV-2 (COVID-19) pandemic. The current literature on the impact of work environment on IBD and IBS symptoms is limited, particularly during the current pandemic.

Objective

This study aims to analyze how the pandemic impacted patients with IBS and IBD in the setting of staying home versus working outside the home.

Methods

After Institutional Review Board (IRB) approval, a retrospective review of 245 patients with IBS and IBD who followed with our gastroenterology clinic in the past year was performed. Patients were asked about symptoms including, but not limited to, worsening diarrhea, constipation, and abdominal pain. Pearson’s chi-squared test was used for analysis.

Results

Of the 245 patients in our study, 67 had IBS, 166 had IBD, and 12 had both. The male-to-female ratio was 1:1.4. A total of 136 (55.5%) patients worked from home during the pandemic, while 109 (44.5%) patients worked outside. Eighty-three patients working from home reported no change in symptoms, 35 reported worsening symptoms, and 18 reported an improvement in symptoms. Sixty-eight patients working outside the home reported no change in symptoms, 26 reported worsening symptoms, and 15 reported improvements. Working outside the home had a statistically significant relationship with COVID-19 infection. Thirty patients were infected, of which 22 (73.3%) worked outside the home (p=0.01). Overall, 203 (82.8%) patients received the vaccine, and only 14 of these patients reported worsening gastrointestinal (GI) symptoms one week after receiving the vaccine. Comparable results were seen after dividing the data into cohorts of IBS and IBD patients. Of the patients with IBD staying at home, 15.9% had depression (p=0.01).

Conclusion

Most patients had symptoms at baseline. There was no statistically significant correlation between change in symptoms and work settings. Patients were less likely to be infected with COVID-19 while staying home. Our patient population showed a high vaccination rate of 82.9% as compared to the national average of 59.2% (source: Centers for Disease Control and Prevention (CDC)). Only 5.7% of the patients reported new or worsening gastrointestinal symptoms in the week following vaccination. The limitations of the study included its retrospective design and poor correlation in general between symptoms and disease activity in IBD patients.

## Introduction

Ulcerative colitis (UC) and Crohn’s disease (CD) are collectively grouped under the umbrella term inflammatory bowel disease (IBD). Both UC and CD are experienced globally with an increasing incidence [1]. The etiology of IBD is thought to be multifactorial, involving a complex interaction between genetic, environmental, microbial, and immunologic factors [2]. Inflammation, ulcerations, fistulas, abscesses, and stricturing can be seen on colonoscopy [3]. Irritable bowel syndrome (IBS) is also chronic in nature but is defined as a continuous or remittent functional gastrointestinal (GI) disorder resulting in a disturbance of bowel function without gross findings [4]. The pathophysiology of IBS is not fully understood and involves complex interactions.

Although IBD and IBS are different in many aspects, as in the overall severity of the disease, they are also akin to one another. Both may present with abdominal pain, bloating, diarrhea, and watery stools. Genetic susceptibility, external environment, intestinal microbial flora, and intestinal immune disruption play roles in both [5]. Environmental and psychological factors such as stress, depression, and negative life events may also serve as predisposing triggers for IBD/IBS [6]. When exposed to a stressful environment, alterations in the brain-gut axis may exacerbate a broad array of gastrointestinal disorders including breakthrough episodes of IBS and/or flares of IBD [7].

Studies have shown that among IBS and IBD patients, stress can be associated with adverse outcomes such as increased frequency of IBS/IBD flares [4,8]. However, very little research has been performed to analyze if there is such a link between IBS/IBD patients and increased stress levels during the COVID-19 pandemic. This retrospective study aimed to analyze the impact of the COVID-19 pandemic on patients with a known diagnosis of IBS and/or IBD with a special focus on the association between stress and IBS/IBD symptomatology when working from home versus when not working from home.

## Materials and methods

This is a retrospective, electronic medical record chart review performed on 700 patients who followed with Albany Medical Center Gastroenterology Clinic up to one year prior to conducting the study. Patients have been assigned the diagnosis of IBD based on endoscopic and histologic or radiologic findings in the setting of IBD-related symptomatology. IBS has been diagnosed based on the Rome IV criteria. Patients suspected of having IBS also undergo extensive evaluation and diagnostic procedures to rule out other causes resulting in their symptoms prior to being diagnosed with IBS. For the purposes of this study, the diagnosis was confirmed through chart review and by asking the patients to affirm their assigned diagnosis. The study was approved by the Institutional Review Board (IRB) of Albany Medical Center. The authors provided a request to the IRB for the initiation of the current study by explaining the goals of the study, the methods that will be used to extract the information, the population that will be under investigation, and how protected health information will be kept safe. After an extensive review, this study was approved (InfoEd number 6156). The current study was conducted between August 2021 and October 2021. Patients were contacted via telephone, and after obtaining consent, a standard questionnaire was used to collect data. Questions that were asked included confirmation of the underlying IBS/IBD type, patients’ employment status and work-related environment, any change in their symptomatology since the start of the pandemic, whether they were infected by COVID-19 and were diagnosed with depression and/or anxiety by a provider (the current study did not utilize a scoring system for depression diagnosis), and whether they had any disease flares that led to hospitalization in the period under investigation. Patients under the age of 18 years were excluded from the study. The current study did not use a standardized scoring system to measure the change in patients’ symptomatology; rather, the study attempted to investigate how patients were evaluating their average daily symptoms when compared to that of prior to the pandemic (stable, worse, or improved).

The primary outcomes included the change in IBS and IBD symptoms during the COVID-19 pandemic ranging from January 2020 to October 2021 based on patients’ responses in our standardized questionnaire (no change, worsening, or improvement). The secondary outcomes covered the investigation of depression and/or anxiety rates in this study population and whether these rates were affected during the pandemic. Changes in IBS and IBD symptomatology after COVID-19 vaccine administration were also examined. Finally, this study’s secondary outcomes also included the investigation of hospital admission rates during the COVID-19 pandemic for the IBS/IBD population and whether there was a difference between patients working from home versus those working away from home.

Statistical analysis was performed using Statistical Package for the Social Sciences (SPSS) version 22 (IBM SPSS Statistics, Armonk, NY, USA). Frequency tables, chi-squared tests, Fisher’s exact test, and cross-tabulations were used to analyze any correlation between variables under investigation and the outcomes of the study. A p-value of <0.05 was considered statistically significant.

## Results

Patient population

Of the 700 total patients contacted, 245 patients participated in the study. The age ranged from 18 to 91 years. Of the patients, 100 were male and 145 were female. A total of 136 (55.5%) patients worked from home, while 109 (44.4%) patients worked outside the home. Overall, 145 (59.2%) patients were employed, 86 (35.1%) were unemployed, and 14 (5.7%) were retired. Table 1 shows different demographic variables and corresponding frequencies for all patients included in the study.

**Table 1:** Demographics, primary outcomes, and secondary outcomes for all 245 patients included in the study. COVID-19: coronavirus disease 2019, GI: gastrointestinal

Variables	All patients (n=245)		
	Home (n=136)	Outside (n=109)	p-value
Gender			0.35
Male	52 (38.2%)	48 (44.4%)	
Female	84 (61.8%)	61 (56%)	
Symptomatic change			0.73
Baseline or improvement	101 (74.3%)	83 (76.1%)	
Worsening symptoms	35 (25.7%)	26 (23.9%)	
Vaccination	110 (80.9%)	93 (85.3%)	0.36
Anxiety related to COVID-19	37 (27.2%)	22 (20.2%)	0.2
COVID-19 infection	8 (5.9%)	22 (20.2%)	0.001
Depression	35 (25.7%)	18 (16.5%)	0.08
Anxiety	40 (29.4%)	30 (27.5%)	0.74
Other GI diseases	34 (25%)	25 (22%)	0.31
Hospital admission/disease flare	15 (11%)	16 (14.7%)	0.39
New or worsening GI symptoms after vaccination	10 (7.4%)	4 (3.7%)	0.04

Primary outcomes

The study showed that 67 (27.3%) patients had IBS, while 166 (67.8%) patients had IBD. Twelve (4%) patients were diagnosed with both IBD and IBS. A total of 88 patients with IBD worked from home, and 78 patients with IBD worked outside their homes. Of these 88 patients working from home, 71 (81.7%) patients showed improvement or baseline symptoms, while 17 (19.3%) patients reported worsening of their symptoms including, but not limited to, abdominal pain, diarrhea, hematochezia, tenesmus, and weight loss. Among the 78 patients with IBD working outside the home, 62 (79.5%) patients were at baseline, and 16 (20.5%) patients showed worsening IBD symptoms. There was no statistically significant difference between symptomatic change and work environment for IBD patients (p=0.83). There were 67 patients with IBS in our study. Of these, 42 worked from home, while 25 worked outside of their homes. A total of 24 (57.1%) IBS patients working from home had symptoms at baseline, while 18 (42.9%) had worsening symptoms. Of the IBS patients working outside the home, 18 (72%) had symptoms at baseline and seven (28.0%) had worsening symptoms. Overall, the majority of IBS and IBD patients had improvement or baseline symptoms regardless of their employment status or work environment (home versus outside). However, the results were not statistically significant for the differences observed. The demographics and results for IBD and IBS patients can be seen in Table 2 and Table 3, respectively.

**Table 2:** Demographics and various variables compared among IBD patients working from home versus working outside of their homes with respective p-values. IBD: inflammatory bowel disease, COVID-19: coronavirus disease 2019, GI: gastrointestinal

Variables	Patients with IBD (n=166)		
	Home (n=88)	Outside (n=78)	p-value
Gender			0.64
Male	42 (47.7%)	40 (51.3%)	
Female	46 (52.3%)	38 (48.7%)	
Symptomatic change			0.83
Baseline or improvement	71 (81.7%)	62 (79.5%)	
Worsening symptoms	17 (19.3%)	16 (20.5%)	
Vaccination	69 (78.4%)	66 (84.6%)	0.3
Anxiety related to COVID-19	21 (23.9%)	15 (19.2%)	0.47
COVID-19 infection	5 (5.7%)	16 (20.5%)	0.004
Depression	14 (15.9%)	3 (3.8%)	0.01
Anxiety	14 (15.9%)	12 (15.4%)	0.92
Other GI diseases	16 (18.1%)	9 (11.5%)	0.21
Hospital admission/disease flare	10 (11.4%)	12 (15.4%)	0.44
New or worsening GI symptoms after vaccination	6 (6.8%)	2 (2.6%)	0.22

**Table 3:** Demographics and various variables compared among IBS patients working from home versus working outside of their homes with respective p-values. IBS: irritable bowel syndrome, COVID-19: coronavirus disease 2019, GI: gastrointestinal

Variables	Patients with IBS (n=67)		
	Home (n=42)	Outside (n=25)	p-value
Gender			0.46
Male	10 (23.8%)	8 (32%)	
Female	32 (76.2%)	17 (68%)	
Symptomatic change			0.22
Baseline or improvement	24 (57.1%)	18 (72%)	
Worsening symptoms	18 (42.9%)	7 (28%)	
Vaccination	35 (83.3%)	21 (84.4%)	0.94
Anxiety related to COVID-19	15 (35.7%)	5 (20%)	0.17
COVID-19 infection	3 (7.1%)	5 (20%)	0.11
Depression	19 (45.2%)	14 (56%)	0.39
Anxiety	23 (54.8%)	15 (60%)	0.67
Other GI diseases	17 (40.4%)	13 (52%)	0.35
Hospital admission/disease flare	5 (11.9%)	2 (8%)	0.61
New or worsening GI symptoms after vaccination	4 (9.5%)	2 (8%)	0.71

Secondary outcomes

The study revealed 14 (15.9%) home-working IBD patients reporting newly diagnosed depression, with only three (3.8%) outside-working patients having similar issues (p=0.01). Conversely, 19 (45.2%) IBS patients working from home reported newly diagnosed depression, while 14 (56%) IBS patients working outside reported depression (p=0.39). Overall, 40 (29.4%) home-working patients suffered from anxiety, with no significant statistical difference from the 30 (27.5%) outside-working patients reporting anxiety (p=0.74). COVID-19 infection was reported in eight (5.9%) patients working from home and in 22 (20.2%) patients working outside (p=0.01). After vaccination, overall, 10 (7.4%) IBD/IBS patients working from home reported a change in their IBS/IBD symptoms compared to just four (3.7%) patients working outside (p=0.04). However, the results were statistically not significant in IBD and IBS subgroup analysis (p=0.22 and p=0.71, respectively). A summary of the measured secondary outcomes can be seen in Table 2 and Table 3.

## Discussion

It has been noted that during the COVID-19 pandemic, stress levels among individuals have increased [4,8,9]. Stress by itself can lead to multiple changes in the gastrointestinal tract, including changes in the bowel’s permeability, motility, and sensitivity [7]. Increased stress levels during the pandemic era are particularly of concern in the subpopulation of individuals diagnosed with IBD and/or IBS because stress is a known factor to instigate IBS/IBD symptomatology [4,8-10]. Interestingly, during the pandemic, it was also reported that IBD patients may have been more fearful about COVID-19 infection compared to other individuals [11]. As such, some studies suggested screening specific IBD patients (e.g., females or those aged 50-70 years old), due to their high risk of developing anxiety during the pandemic, which in turn may lead to vicious cycles of anxiety and higher disease activity [11,12].

In our study, when comparing the IBS/IBD symptomatology and work setting, most IBD and IBS patients reported improvement or “at baseline” symptoms during the period in question, the COVID-19 pandemic. Interestingly, other large studies analyzing IBS and IBD were in line with these findings. A large study involving IBS patients indicated symptomatic improvement or “at baseline” symptoms during the pandemic (although IBS patients had poorer psychological well-being compared to non-IBS patients), referring to this observation as the “COVID-19 IBS paradox.” Multiple factors such as occupational stress and burnout may lead to the worsening of IBS, improved by the work from home policy during the pandemic, and may have contributed to this paradox [13-18]. Hence, it was hypothesized that improved control over the work schedule is associated with improvement or at least non-worsening of IBS symptomatology. The importance of being in control and how strongly it may influence IBS symptoms is also evident when considering that patients with limited knowledge of COVID-19, who were complying with the measures only because of fear of the consequences, had higher chances of symptom deterioration [18]. Social support can also affect IBS symptoms and as such may play a big role in the discrepancies noted in the literature. In general, better social support during the pandemic is associated with non-worsening of IBS symptoms, while lack of support has been linked with clinical deterioration [18-20]. Another great example of how social support positively influences IBS symptomatology is that unmarried IBS patients exhibit more severe symptoms [19]. This study did not reveal any change in IBS patients’ symptomatology when different working environments were compared.

Similar results have been noted among IBD patients. Although some studies indicate lower quality of life as an effect of the pandemic, other investigators support that despite recent barriers in IBD patients’ healthcare, such as delays in procedures, appointments, and drug inaccessibility, the quality of life has not changed significantly [21-24]. For example, in the Netherlands, investigators reported worsening in quality of life and gastrointestinal symptomatology, but a tertiary care center in Saudi Arabia did not reveal worsening in IBD patients’ quality of life [23,24]. The insignificant changes in symptoms observed by some studies could also be attributed to the fact that IBD patients may have been more socially isolated even prior to the pandemic due to the overwhelming symptomatology they usually suffer from. This, in combination with the increased social support that some patients had, may lead to the grossly unchanged symptoms described by some studies [21]. Concerns were also raised due to the high utilization of telemedicine (e.g., mobile applications or communication technologies) during the pandemic and that this might worsen IBD symptoms or psychology. However, no difference in psychological outcomes was noted when telemedicine was compared to standard care [11,21,25]. Our study again revealed that the majority of IBD patients had “at baseline” or improved symptoms. Moreover, the current study failed to show the impact of the working environment on IBD patients during the pandemic.

Alternatively, our study revealed a clear association between depression rates and working environments. Specifically, IBD patients working from home were found to have significantly higher rates of depression when compared to those working outside the home. Such a relationship was not observed in IBS patients. Other studies report vicious cycles of worsening psychological status and symptomatic worsening in IBD patients [11]. Especially, females and patients between 50 and 70 years old may be prone to worse levels of depression and should be screened [12].

Finally, COVID-19 vaccination was associated with new or worsening gastrointestinal symptoms in IBS/IBD patients who were working from home. However, the relationship was not validated in the subgroup analysis.

The current study has a multitude of limitations, including the retrospective nature of the study, the poor correlation between symptomatology and disease activity, the extraction of information through a standardized questionnaire for a period of almost two years leading to possible inaccurate recall by the respondents or misunderstanding, the study population that was not randomly selected, and the response rate in our study group that was low (245/700), which all played a role in forming the limitations of the current study. Moreover, the lack of a standardized scoring system to measure patients’ symptom changes may lead to more subjective results.

## Conclusions

Although stress levels have been noted to be higher during the pandemic and stress is a known risk factor for IBS/IBD symptom worsening, the current study revealed that most IBD/IBS patients under investigation reported symptoms at baseline. Multiple studies have explored the pandemic’s effect on this population, revealing conflicting results. Despite the general consensus that the psychosocial well-being of IBD/IBS patients has been negatively affected, it is questionable how hard this influenced the symptomatology of these patients. This is best depicted by the “COVID-19 IBS paradox” described in the literature, which refers to stable symptoms in IBS patients, despite their poorer psychological well-being described in other studies during the pandemic. The IBD population has also been examined during the pandemic era with contradictory results regarding their quality of life and the effect the pandemic had on their symptoms. The routine care of these patients was heavily disrupted during the pandemic, and telemedicine was implemented to compensate for this disruption. As such, multiple studies raised concerns that IBD patients’ quality of life may worsen; however, it is controversial whether this occurred.

Several hypotheses have been proposed to explain this observation, and apparently, social support is key, with patients having stronger support by their social cycle and experiencing a superior health-related quality of life during the pandemic. It is plausible that the varying levels of social support available to the IBS/IBD population played a big role in the control of their symptoms, leading to the aforementioned inconsistent observations in the literature. Moreover, social isolation during the pandemic may have not affected largely the IBD population, since many IBD patients have been more isolated, even prior to the pandemic, compared to the general population due to their symptoms.

The work environment may also contribute to the psychosocial well-being of IBS/IBD patients. For example, working from home could potentially lead to fewer interactions and less social support and as such to worse psychosocial well-being and worsening symptomatology. On the other hand, though, working from home could lead to better control over patients’ own daily schedule and as such to better psychological outcomes. Working outside may lead to more social interactions but will have the opposite effect on the schedule management. Moreover, it has been proposed that patients who used to work outside could become more anxious about COVID-19 infection, leading to higher stress levels and potentially worsening of IBD/IBS symptomatology. The current study revealed no association between symptomatic changes and the work environment in the IBS/IBD population. However, IBD patients working from home had higher rates of depression. In general, more studies are needed to reveal how the COVID-19 pandemic affected the psychosocial well-being of the IBD/IBS population and/or whether their symptoms also changed. Moreover, studies are also required to elucidate whether and how working in specific environments benefits IBS/IBD patients.
